# Advances in the Study of Mirror Neurons and Their Impact on Neuroscience: An Editorial

**DOI:** 10.7759/cureus.61299

**Published:** 2024-05-29

**Authors:** Jitendra Patel

**Affiliations:** 1 Physiology, Gujarat Medical Education and Research Society Medical College, Vadnagar, IND

**Keywords:** patient, social, cognition, neuroscience, mirror neurons

## Abstract

The mirror neurons are complex neuronal circuits in the brain, and they respond to the actions that we observe in others. The mirror neurons constitute a revolutionary discovery in the field of neuroscience that has not only reshaped our understanding of social cognition and empathetic behavior but also bridged gaps in our comprehension of the human brain's intricate workings. This article aims to distill the crux of these groundbreaking discoveries and their transformative ramifications regarding our perception of human interactions and the advancement of neurorehabilitation techniques. The integration of non-invasive and patient-centric therapies into clinical practice underscores the immense potential that research on mirror neurons holds in enhancing patient outcomes and quality care. Research in mirror neurons will contribute significantly to the field of neuroscience, specifically neurorehabilitation.

## Editorial

The mirror neurons are specialized neurons that “mirror” the actions and behaviors of others and they play a crucial role in the realms of cognition, language, and empathy. The mirror neurons were first discovered in macaques. These specialized neurons are activated both when an individual performs an action and when they observe someone else performing the same action, thus linking action observation with action execution. It has been reported that different regions of the brain are involved in this process. The latest research shows that many cortical structures are also part of this mirror neuron system (MNS).

Mirror neurons play a crucial role in neuroscience by enabling individuals to understand and imitate the actions of others. This mirroring mechanism is believed to underlie processes such as empathy, imitation, social cognition, and even language development. Research on mirror neurons has provided insights into how the brain processes and interprets social cues, how it contributes to motor learning and supports social interactions and understanding. The study of these neurons continues to advance our understanding of human behavior, communication, and the neural basis of social interaction.

It is worth delving deeper into the remarkable strides made in current research, such as the innovative strides in mirror therapy, as documented in the study by Vér and Vér [1]. This cutting-edge therapy, leveraging the principles of neuroplasticity and kinesthetic memory, has shown great promise in the realm of medical treatment, targeting conditions such as hemiplegia after stroke, gait recovery after stroke, improvement of finger coordination in Parkinson's patients, reduction of phantom limb pain after amputations, and chronic pain from hand osteoarthritis, fibromyalgia, and complex regional pain syndrome [1]. The device we focus on in the present article constitutes a new constructive variant of the mirror box, which offers a much more efficient method of applying mirror therapy in various conditions, both at the level of the upper and lower limbs [1].

Research on mirror neurons has significantly advanced in recent years, shedding light on their role in understanding others' actions and intentions. Studies have explored the molecular identity of mirror neurons through single-cell transcriptomics, delved into the temporal dynamics and stimuli-dependent modulation of mirror neuron effects, and investigated the non-shared coding of observed and executed actions in macaque ventral premotor mirror neurons. These advancements have enhanced our understanding of how mirror neurons contribute to processes such as self-awareness, emotional contagion, and motor learning, emphasizing their importance in social cognition and behavior.

Mirror neurons play a significant role in therapy by enabling interventions that leverage their functioning to promote motor recovery, pain management, and cognitive rehabilitation. Mirror therapy, utilizing the mirror neuron system, has shown promise in various conditions such as stroke rehabilitation, phantom limb pain, and neurorehabilitation. By engaging mirror neurons through activities like mirror visual feedback, imitation, and action observation, therapists can facilitate motor learning, enhance self-awareness, and improve functional outcomes in patients undergoing rehabilitation. The application of mirror neuron principles in therapy underscores their potential to enhance motor skills, alleviate pain, and foster neuroplasticity in diverse clinical populations.

Mirror therapy works by utilizing the neural phenomenon of mirror neurons in the brain. During mirror therapy, a patient performs movements with their unaffected limb while observing the mirror reflection of that limb in place of the affected limb. This visual illusion created by the mirror gives the impression that the affected limb is moving correctly. The mirror neuron system in the brain is activated, causing the brain to perceive the reflected limb as the affected limb moving. This visual feedback helps rewire the brain's neural pathways, leading to improvements in motor function, pain management, and neuroplasticity. The synchronization between the visual feedback and motor execution from the unaffected limb can facilitate motor learning and enhance rehabilitation outcomes.

Equally noteworthy is the confluence of mirror neuron exploration with the latest advancements in technology, as illustrated by Chen and Fang [2]. The evolution of implantable neural probe technologies is opening up new horizons in the study of neural circuitry. These state-of-the-art devices are set to revolutionize our insights into the brain's function by offering high-resolution recording and modulation of neural activity. The prospects of these probes in advancing our comprehension of empathy and social cognition, underpinned by the activity of mirror neurons, are a thrilling frontier in neuroscience research [2]. Furthermore, the methodological progress in analyzing organoid networks, as explored by Chen et al. [3], offers evidence of the growing potential of neuroscience. The employment of organoid models and change point algorithms opens up new avenues for understanding the intricacies of brain network dynamics. Such sophisticated techniques could pave the way for earlier detection of neurological disorders and provide a more nuanced understanding of the role and behavior of mirror neurons within these elaborate networks [3].

The practical implications of this body of research cannot be overstated. As we forge ahead, we must ensure that these scientific breakthroughs translate into tangible therapeutic tools that are both accessible and cost-effective for the wider population. The application of multifractal analysis in the early detection of neurodegenerative diseases, such as Alzheimer's, as posited by Ahmad et al. [4], exemplifies the kind of innovative approaches that could have a substantial impact on diagnostic and treatment strategies [4].

In light of the compelling advancements discussed so far, it is clear that the intersection of neuroscience, technology, and clinical application is an area ripe with opportunities for transformative research. The use of mirror neuron principles in the development of novel therapies, such as those for Parkinson's disease as described by Pawłowski et al. [5], further accentuates the vital role of translational research in improving the lives of individuals affected by such debilitating conditions [5].

Modern treatment approaches based on mirror neurons have gained traction in various neurological and neurorehabilitation fields. Recent research has explored the role of defective mirror neurons in autism spectrum disorder. The efficacy of current treatment approaches for autism spectrum disorder, which may involve interventions targeting mirror neuron functioning, needs to be assessed. In Parkinson's disease, advancements in treatment methods have been explored, potentially leveraging insights from mirror neuron exploration. By understanding the interaction between mirror neuron dysfunction and motor symptoms in conditions like Parkinson's disease, tailored treatment approaches can be developed to address specific deficits. Furthermore, in the realm of neurodegenerative diseases such as spinal muscular atrophy, by harnessing the potential of mirror neuron-based interventions, clinicians aim to augment motor function and quality of life for individuals affected by spinal muscular atrophy. These developments underscore the evolving landscape of modern treatment strategies that integrate knowledge of mirror neurons to inform personalized interventions and neurorehabilitation approaches across various neurological conditions.

The scientific community should vigorously support this transformative idea in the field of neuroscience. Mirror neurons have the potential to influence practical therapeutic approaches beyond academic research boundaries and significantly elevate the standard of care for individuals who struggle with neurological disorders. We eagerly anticipate further research on mirror neurons, which will continue to illuminate these pivotal advancements in neuroscience.
